# Methyl 4-(piperidin-1-ylcarbon­yl)benzoate

**DOI:** 10.1107/S1600536810021720

**Published:** 2010-06-16

**Authors:** R. M. D. Ávila, I. M. R. Landre, T. E. Souza, M. P. Veloso, A. C Doriguetto

**Affiliations:** a Laboratório de Fitoquímica e Química Medicinal, Instituto de Ciências Exatas, Universidade Federal de Alfenas, Alfenas, MG 37130-000, Brazil; b Laboratório de Cristalografia, Instituto de Ciências Exatas, Universidade Federal de Alfenas- Unifal-MG, Alfenas, MG 37130-000, Brazil

## Abstract

In the title compound, C_14_H_17_NO_3_, the piperidine ring has a chair conformation and an intra­molecular C—H⋯O inter­action stabilizes the mol­ecular conformation. In the crystal, weak inter­molecular C—H⋯O inter­actions occur.

## Related literature

For Pd(0)-catalysed carbonyl­ation of aryl halides, see: Jia & Morris (1991 ▶); Stille & Wong (1975 ▶); Magerlein, *et al.* (2001 ▶); Zhao *et al.* (2008 ▶). For procedural modifications for carbonyl­ation reactions, see: Lagerlund & Larhed (2006 ▶). For the preparation of other piperidine derivatives, see Lima *et al.* (2002 ▶). For bond-length data, see: Allen *et al.* (1987 ▶).
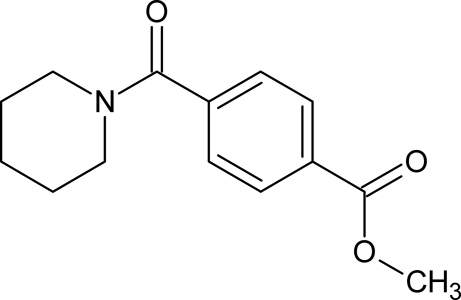


## Experimental

### 

#### Crystal data

C_14_H_17_NO_3_
*M* *_r_* = 247.29
Triclinic, 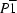

*a* = 5.879 (5) Å
*b* = 9.693 (5) Å
*c* = 12.190 (5) Å
α = 69.684 (5)°
β = 82.535 (5)°
γ = 77.502 (5)°
*V* = 634.9 (7) Å^3^
*Z* = 2
Mo *K*α radiation
μ = 0.09 mm^−1^
*T* = 150 K
0.05 × 0.05 × 0.05 mm

#### Data collection

Oxford Diffraction Xcalibur Atlas Gemini Ultra diffractometer
4958 measured reflections
2958 independent reflections
2036 reflections with *I* > 2σ(*I*)
*R*_int_ = 0.033

#### Refinement

*R*[*F* ^2^ > 2σ(*F* ^2^)] = 0.044
*wR*(*F* ^2^) = 0.122
*S* = 1.01
2958 reflections
164 parameters
H-atom parameters constrained
Δρ_max_ = 0.25 e Å^−3^
Δρ_min_ = −0.26 e Å^−3^

### 

Data collection: *CrysAlis PRO* (Oxford Diffraction, 2010 ▶); cell refinement: *CrysAlis PRO*; data reduction: *CrysAlis PRO*; program(s) used to solve structure: *SIR92* (Altomare *et al.*, 1999 ▶); program(s) used to refine structure: *SHELXL97* (Sheldrick, 2008 ▶); molecular graphics: *ORTEP-3 for Windows* (Farrugia, 1997 ▶); software used to prepare material for publication: *WinGX* (Farrugia, 1999 ▶).

## Supplementary Material

Crystal structure: contains datablocks global, I. DOI: 10.1107/S1600536810021720/zs2043sup1.cif

Structure factors: contains datablocks I. DOI: 10.1107/S1600536810021720/zs2043Isup2.hkl

Additional supplementary materials:  crystallographic information; 3D view; checkCIF report

## Figures and Tables

**Table 1:** Hydrogen-bond geometry (Å, °)

*D*—H⋯*A*	*D*—H	H⋯*A*	*D*⋯*A*	*D*—H⋯*A*
C5—H5*A*⋯O1	0.97	2.33	2.750 (3)	105
C14—H14*B*⋯O2^i^	0.96	2.56	3.436 (4)	153

Symmetry code: (i) 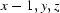.

**Fig. 1.**
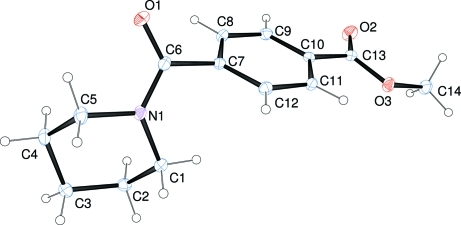
The structure of the title compound showing the atom-numbering scheme. Displacement ellipsoids are drawn at the 50% probability level.

**Fig. 2.**
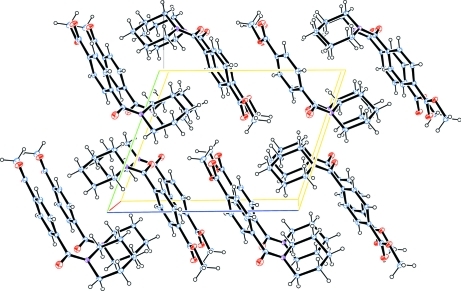
The crystal packing of (I).

## supplementary crystallographic information

### Comment

There are many methods documented for the synthesis of a wide range of aromatic carboxylic acid derivatives, including benzamides. These compounds can be prepared using palladium(0)-catalyzed carbonylation of aryl halides with various nucleophiles (Zhao *et al.*, 2008; Margerlein *et al.*, 2001; Stille & Wong, 1975). The aminocarbonylation reaction of aryl halides achieved using the commercially available preligand [(^t^Bu)_3_PH]BF_4_ as the key component in combination with Herrmann's palladacycle as the Pd source (Jia & Morris, 1991). In other studies procedures employing Mo(CO)_6_ as a carbon monoxide releasing reagent, together with the use of controlled microwave irradiation as the energy source have been used to overcome the problems of introducing a gaseous reactant in small-scale high-speed protocols (Lagerlund & Larhed, 2006). In addition to other methods for obtaining derivatives of aromatic carboxylic acids, methyl 4-(piperidine-1-carbonyl)benzoate, C_14_H_17_N_1_O_3_ (I) was prepared from 4-(methoxycarbonyl)benzoic acid in excellent yield, exploring classical methodology, using thionyl chloride as the more electrophilic acid chloride, followed by treatment with piperidine in the presence of chloroform, at room temperature (Lima *et al.*, 2002). The study of this reaction showed that it could be controlled by the stoichimetric and reaction conditions, making the reaction of the piperidine with acyl chloride more favoured than with the ester group, by the use of an easy and convenient method.

In the structure of the title compound (Fig. 1) all bond lengths and angles are in agreement with literature values (Allen *et al.*, 1987). The aromatic ring and the ester are close to planar [C9–C10–C13–O3, -171.21 (12)°; C10–C13–O3–C14, 175.10 (11)°], whereas the carbonyl group is twisted out of the plane of the ring [C12–C7–C6–O1, 122.57 (15)°]. The piperidine ring has the more energetically favored boat conformation, with an intramolecular C5—H···O(carbonyl) interaction [C···O, 2.750 (3) Å] which stabilizes the molecular conformation. As expected, the supramolecular structure has no formal intermolecular hydrogen bonds (Fig. 2).

### Experimental

A solution of 0.50 g of 4-(methoxycarbonyl)benzoic acid in 15 ml of chloroform, 0.30 ml of freshly distilled thionyl chloride and a catalytic amount of dimethylformamide was stirred under reflux for 1 h. After this time, the solvent was carefully evaporated at reduced pressure and a solution of 2.78 mmol of piperidine and 0.78 ml of triethylamine in 10 ml of chloroform was added. The reaction mixture was stirred for 30 min at room temperature, after which 10 ml of saturated sodium carbonate aqueous solution was added and the mixture extracted with chloroform (3x15 ml). The organic layer was separated, washed with water, rewashed with brine and dried over anhydrous sodium sulfate. The solvent was evaporated at reduced pressure after which the compound was purified by column chromatography using Merck Silica Gel 60 (0.040–0.063 mm) and a mixture of hexane/ethyl acetate (8:2,V/V) as eluent (0.56 g, 82%). Crystals suitable for X-ray diffraction were grown from a mixture of hexane/ethyl acetate (8:2, V/V). IR (KBr, cm^-1^): ν 1724, 1680, 1436, 1276, 1114. ^1^H NMR (200 MHz, CDCl_3_, p.p.m.): δ 1.27 (m, 2H), 1.70 (m, 2H), 3.33 (m, 2H), 3.74 (m, 2H), 3.95 (s, 3H), 7.47 (d, ^3^ J = 8.27 Hz), 8.09 (d, ^3^ J = 8.17). ^13^C NMR (200 MHz, CDCl_3_, p.p.m.): δ 24.5, 25.6, 26.5, 42.2, 51.2, 52.6, 126.7, 129.8, 130.8, 140.9, 166.4, 169.2.

### Refinement

The H atoms were located from the difference electron density synthesis and allowed to ride on their parent atoms, with C—H(aromatic) = 0.95 Å and C—H(aliphatic) = 0.97 Å and *U*_iso_(H)= 1.5*U*_eq_ for methyl H atoms or 1.2*U*_eq_ for the remaining H atoms.

### Crystal data

**Table:**

C_14_H_17_NO_3_	*Z* = 2
*M**_r_* = 247.29	*F*(000) = 264
Triclinic, *P*1	*D*_x_ = 1.294 Mg m^−^^3^
Hall symbol: -P 1	Mo *K*α radiation, λ = 0.71073 Å
*a* = 5.879 (5) Å	Cell parameters from 2414 reflections
*b* = 9.693 (5) Å	θ = 3.3–29.4°
*c* = 12.190 (5) Å	µ = 0.09 mm^−^^1^
α = 69.684 (5)°	*T* = 150 K
β = 82.535 (5)°	Prism, colourless
γ = 77.502 (5)°	0.05 × 0.05 × 0.05 mm
*V* = 634.9 (7) Å^3^	

### Data collection

**Table:**

Oxford Diffraction Xcalibur Atlas Gemini Ultra diffractometer	*R*_int_ = 0.033
Detector resolution: 10.4186 pixels mm^-1^	θ_max_ = 29.4°, θ_min_ = 3.3°
ω scans	*h* = −7→7
4958 measured reflections	*k* = −11→12
2958 independent reflections	*l* = −13→16
2036 reflections with *I* > 2σ(*I*)	

### Refinement

**Table:**

Refinement on *F*^2^	0 restraints
Least-squares matrix: full	H-atom parameters constrained
*R*[*F*^2^ > 2σ(*F*^2^)] = 0.044	*w* = 1/[σ^2^(*F*_o_^2^) + (0.0676*P*)^2^] where *P* = (*F*_o_^2^ + 2*F*_c_^2^)/3
*wR*(*F*^2^) = 0.122	(Δ/σ)_max_ < 0.001
*S* = 1.01	Δρ_max_ = 0.25 e Å^−^^3^
2958 reflections	Δρ_min_ = −0.26 e Å^−^^3^
164 parameters	

### Special details

**Table:**

Geometry. All e.s.d.'s (except the e.s.d. in the dihedral angle between two l.s. planes) are estimated using the full covariance matrix. The cell e.s.d.'s are taken into account individually in the estimation of e.s.d.'s in distances, angles and torsion angles; correlations between e.s.d.'s in cell parameters are only used when they are defined by crystal symmetry. An approximate (isotropic) treatment of cell e.s.d.'s is used for estimating e.s.d.'s involving l.s. planes.

### Fractional atomic coordinates and isotropic or equivalent isotropic displacement parameters (Å^2^)

**Table:**

	*x*	*y*	*z*	*U*_iso_*/*U*_eq_	
O3	0.73762 (16)	0.78416 (10)	0.51830 (8)	0.0285 (2)	
O2	1.10549 (18)	0.65801 (11)	0.54066 (9)	0.0377 (3)	
O1	1.41852 (17)	1.30674 (12)	0.11938 (9)	0.0378 (3)	
N1	1.14709 (19)	1.30851 (12)	0.00274 (9)	0.0232 (3)	
C7	1.1642 (2)	1.13450 (14)	0.20582 (11)	0.0214 (3)	
C11	0.8703 (2)	1.03005 (14)	0.34869 (11)	0.0220 (3)	
H11	0.7179	1.0388	0.3812	0.026*	
C10	1.0327 (2)	0.90031 (14)	0.39573 (11)	0.0211 (3)	
C13	0.9679 (2)	0.76837 (15)	0.49291 (11)	0.0240 (3)	
C4	1.3404 (3)	1.36011 (16)	−0.19405 (12)	0.0285 (3)	
H4A	1.4773	1.2843	−0.167	0.034*	
H4B	1.389	1.439	−0.2616	0.034*	
C8	1.3275 (2)	1.00679 (15)	0.25594 (12)	0.0256 (3)	
H8	1.482	1	0.2262	0.031*	
C12	0.9354 (2)	1.14597 (14)	0.25349 (11)	0.0234 (3)	
H12	0.8259	1.2316	0.2214	0.028*	
C5	1.2352 (3)	1.42408 (15)	−0.09739 (12)	0.0281 (3)	
H5A	1.3527	1.4615	−0.0725	0.034*	
H5B	1.1085	1.5071	−0.1269	0.034*	
C3	1.1643 (3)	1.29141 (15)	−0.22919 (12)	0.0275 (3)	
H3A	1.2388	1.2434	−0.2858	0.033*	
H3B	1.0372	1.3697	−0.266	0.033*	
C2	1.0677 (2)	1.17679 (15)	−0.12294 (11)	0.0260 (3)	
H2A	0.9453	1.1417	−0.1463	0.031*	
H2B	1.1909	1.0915	−0.093	0.031*	
C6	1.2522 (2)	1.25752 (15)	0.10503 (12)	0.0233 (3)	
C9	1.2617 (2)	0.89002 (15)	0.34957 (12)	0.0259 (3)	
H9	1.3711	0.8044	0.3817	0.031*	
C1	0.9701 (2)	1.24338 (16)	−0.02665 (12)	0.0258 (3)	
H1A	0.8338	1.3201	−0.0527	0.031*	
H1B	0.9226	1.1659	0.0424	0.031*	
C14	0.6585 (3)	0.65522 (17)	0.60534 (13)	0.0328 (3)	
H14A	0.7227	0.5674	0.5842	0.049*	
H14B	0.4912	0.6707	0.6088	0.049*	
H14C	0.7094	0.6423	0.6806	0.049*	

### Atomic displacement parameters (Å^2^)

**Table:**

	*U*^11^	*U*^22^	*U*^33^	*U*^12^	*U*^13^	*U*^23^
O3	0.0247 (5)	0.0288 (5)	0.0255 (5)	−0.0082 (4)	0.0020 (4)	−0.0002 (4)
O2	0.0287 (6)	0.0328 (6)	0.0408 (6)	−0.0048 (5)	−0.0073 (5)	0.0027 (5)
O1	0.0341 (6)	0.0479 (6)	0.0359 (6)	−0.0257 (5)	−0.0023 (5)	−0.0085 (5)
N1	0.0275 (6)	0.0235 (6)	0.0219 (6)	−0.0127 (5)	0.0033 (4)	−0.0085 (5)
C7	0.0230 (7)	0.0256 (6)	0.0204 (7)	−0.0095 (5)	0.0002 (5)	−0.0110 (5)
C11	0.0180 (7)	0.0274 (7)	0.0226 (7)	−0.0067 (5)	0.0020 (5)	−0.0102 (6)
C10	0.0228 (7)	0.0254 (7)	0.0184 (6)	−0.0077 (5)	−0.0025 (5)	−0.0088 (5)
C13	0.0234 (7)	0.0286 (7)	0.0215 (7)	−0.0067 (6)	−0.0033 (5)	−0.0083 (6)
C4	0.0320 (8)	0.0276 (7)	0.0240 (7)	−0.0118 (6)	0.0052 (6)	−0.0050 (6)
C8	0.0175 (7)	0.0344 (7)	0.0260 (7)	−0.0073 (6)	0.0012 (5)	−0.0108 (6)
C12	0.0216 (7)	0.0233 (7)	0.0260 (7)	−0.0030 (5)	−0.0020 (5)	−0.0095 (6)
C5	0.0356 (8)	0.0213 (7)	0.0279 (7)	−0.0126 (6)	0.0033 (6)	−0.0064 (6)
C3	0.0311 (8)	0.0294 (7)	0.0221 (7)	−0.0055 (6)	−0.0010 (5)	−0.0090 (6)
C2	0.0286 (8)	0.0279 (7)	0.0251 (7)	−0.0089 (6)	−0.0044 (6)	−0.0099 (6)
C6	0.0214 (7)	0.0252 (7)	0.0259 (7)	−0.0077 (5)	0.0034 (5)	−0.0113 (6)
C9	0.0221 (7)	0.0291 (7)	0.0257 (7)	−0.0020 (6)	−0.0053 (5)	−0.0083 (6)
C1	0.0235 (7)	0.0308 (7)	0.0250 (7)	−0.0103 (6)	−0.0004 (5)	−0.0087 (6)
C14	0.0298 (8)	0.0348 (8)	0.0278 (8)	−0.0132 (6)	0.0000 (6)	0.0008 (6)

### Geometric parameters (Å, °)

**Table:**

O3—C13	1.337 (2)	C4—H4B	0.97
O3—C14	1.4514 (17)	C8—C9	1.3826 (19)
O2—C13	1.2054 (17)	C8—H8	0.93
O1—C6	1.2320 (18)	C12—H12	0.93
N1—C6	1.3496 (18)	C5—H5A	0.97
N1—C1	1.4655 (19)	C5—H5B	0.97
N1—C5	1.4664 (17)	C3—C2	1.5223 (19)
C7—C12	1.391 (2)	C3—H3A	0.97
C7—C8	1.393 (2)	C3—H3B	0.97
C7—C6	1.5113 (18)	C2—C1	1.5213 (19)
C11—C12	1.3861 (18)	C2—H2A	0.97
C11—C10	1.3942 (19)	C2—H2B	0.97
C11—H11	0.93	C9—H9	0.93
C10—C9	1.387 (2)	C1—H1A	0.97
C10—C13	1.4918 (19)	C1—H1B	0.97
C4—C5	1.5191 (19)	C14—H14A	0.96
C4—C3	1.520 (2)	C14—H14B	0.96
C4—H4A	0.97	C14—H14C	0.96
			
C13—O3—C14	115.47 (10)	C4—C5—H5B	109.6
C6—N1—C1	125.92 (11)	H5A—C5—H5B	108.1
C6—N1—C5	119.70 (12)	C4—C3—C2	110.94 (12)
C1—N1—C5	113.67 (11)	C4—C3—H3A	109.5
C12—C7—C8	119.40 (12)	C2—C3—H3A	109.5
C12—C7—C6	123.66 (11)	C4—C3—H3B	109.5
C8—C7—C6	116.86 (12)	C2—C3—H3B	109.5
C12—C11—C10	120.13 (12)	H3A—C3—H3B	108
C12—C11—H11	119.9	C1—C2—C3	111.32 (11)
C10—C11—H11	119.9	C1—C2—H2A	109.4
C9—C10—C11	119.69 (12)	C3—C2—H2A	109.4
C9—C10—C13	118.03 (11)	C1—C2—H2B	109.4
C11—C10—C13	122.26 (12)	C3—C2—H2B	109.4
O2—C13—O3	123.63 (13)	H2A—C2—H2B	108
O2—C13—C10	124.22 (13)	O1—C6—N1	122.56 (12)
O3—C13—C10	112.10 (11)	O1—C6—C7	118.58 (12)
C5—C4—C3	110.68 (12)	N1—C6—C7	118.86 (12)
C5—C4—H4A	109.5	C8—C9—C10	120.14 (12)
C3—C4—H4A	109.5	C8—C9—H9	119.9
C5—C4—H4B	109.5	C10—C9—H9	119.9
C3—C4—H4B	109.5	N1—C1—C2	110.13 (12)
H4A—C4—H4B	108.1	N1—C1—H1A	109.6
C9—C8—C7	120.44 (13)	C2—C1—H1A	109.6
C9—C8—H8	119.8	N1—C1—H1B	109.6
C7—C8—H8	119.8	C2—C1—H1B	109.6
C11—C12—C7	120.15 (12)	H1A—C1—H1B	108.1
C11—C12—H12	119.9	O3—C14—H14A	109.5
C7—C12—H12	119.9	O3—C14—H14B	109.5
N1—C5—C4	110.27 (11)	H14A—C14—H14B	109.5
N1—C5—H5A	109.6	O3—C14—H14C	109.5
C4—C5—H5A	109.6	H14A—C14—H14C	109.5
N1—C5—H5B	109.6	H14B—C14—H14C	109.5
			
C12—C11—C10—C9	2.35 (19)	C5—C4—C3—C2	−54.04 (15)
C12—C11—C10—C13	−175.86 (12)	C4—C3—C2—C1	53.59 (15)
C14—O3—C13—O2	−2.60 (19)	C1—N1—C6—O1	171.43 (13)
C14—O3—C13—C10	175.10 (11)	C5—N1—C6—O1	1.71 (19)
C9—C10—C13—O2	6.5 (2)	C1—N1—C6—C7	−8.12 (19)
C11—C10—C13—O2	−175.28 (13)	C5—N1—C6—C7	−177.84 (11)
C9—C10—C13—O3	−171.20 (11)	C12—C7—C6—O1	122.57 (15)
C11—C10—C13—O3	7.03 (18)	C8—C7—C6—O1	−54.07 (17)
C12—C7—C8—C9	2.31 (19)	C12—C7—C6—N1	−57.86 (18)
C6—C7—C8—C9	179.10 (12)	C8—C7—C6—N1	125.50 (14)
C10—C11—C12—C7	−1.17 (19)	C7—C8—C9—C10	−1.1 (2)
C8—C7—C12—C11	−1.15 (19)	C11—C10—C9—C8	−1.19 (19)
C6—C7—C12—C11	−177.71 (12)	C13—C10—C9—C8	177.09 (12)
C6—N1—C5—C4	112.46 (14)	C6—N1—C1—C2	−112.70 (14)
C1—N1—C5—C4	−58.47 (15)	C5—N1—C1—C2	57.56 (15)
C3—C4—C5—N1	55.55 (15)	C3—C2—C1—N1	−54.21 (15)

### Hydrogen-bond geometry (Å, °)

**Table:**

*D*—H···*A*	*D*—H	H···*A*	*D*···*A*	*D*—H···*A*
C5—H5A···O1	0.97	2.33	2.750 (3)	105
C14—H14B···O2^i^	0.96	2.56	3.436 (4)	153

Symmetry codes: (i) *x*−1, *y*, *z*.
